# Primary Pulmonary Anaplastic Large Cell Lymphoma: A Rare Malignancy and Rare Cause of the Luftsichel Sign

**DOI:** 10.1155/2018/8574642

**Published:** 2018-05-06

**Authors:** Elizabeth Von Ende, Travis Kauffman, Philip A. Munoz, Santiago Martinez-Jiménez

**Affiliations:** ^1^Kansas City University, 1750 Independence Ave, Kansas City, MO 64106, USA; ^2^University of Missouri-Kansas City, 4401 Wornall Rd, Kansas City, MO 64111, USA; ^3^Saint Luke's Hospital of Kansas City, University of Missouri-Kansas City, 4401 Wornall Rd, Kansas City, MO 64111, USA

## Abstract

Primary pulmonary lymphomas are rare with primary pulmonary non-Hodgkin lymphoma accounting for only 0.3% of primary lung neoplasms. Of these, the large majority are made up of marginal zone B-cell lymphoma and diffuse large B-cell lymphoma. We present a case of a very rare primary pulmonary anaplastic large cell lymphoma presenting as the luftsichel sign on chest radiograph. Pertinent imaging and pathology findings are discussed.

## 1. Introduction

Primary pulmonary lymphomas (PPL) are overall rare neoplastic malignancies. Notably primary non-Hodgkin lymphoma (P-NHL) accounts for only 0.3% of primary lung neoplasms and primary pulmonary Hodgkin lymphoma is even rarer. Among P-NHL, marginal zone B-cell lymphoma of mucosa-associated lymphoid tissue and diffuse large B-cell lymphoma are responsible for over 95% of all PPL [1, 2]. Other types of lymphoproliferative processes such as primary lung plasmacytoma and lymphomatoid granulomatosis account for a good proportion of the remaining PPL [3]. Anaplastic large cell lymphoma (ALCL), which most often occurs in lymph nodes and skin, is an exceedingly rare type of PPL [1, 2, 4]. Here we describe a case of primary ALCL of the lung in a patient who made a complete recovery after presenting with complete left upper lobe atelectasis.

## 2. Case Presentation

**Figure 1:**
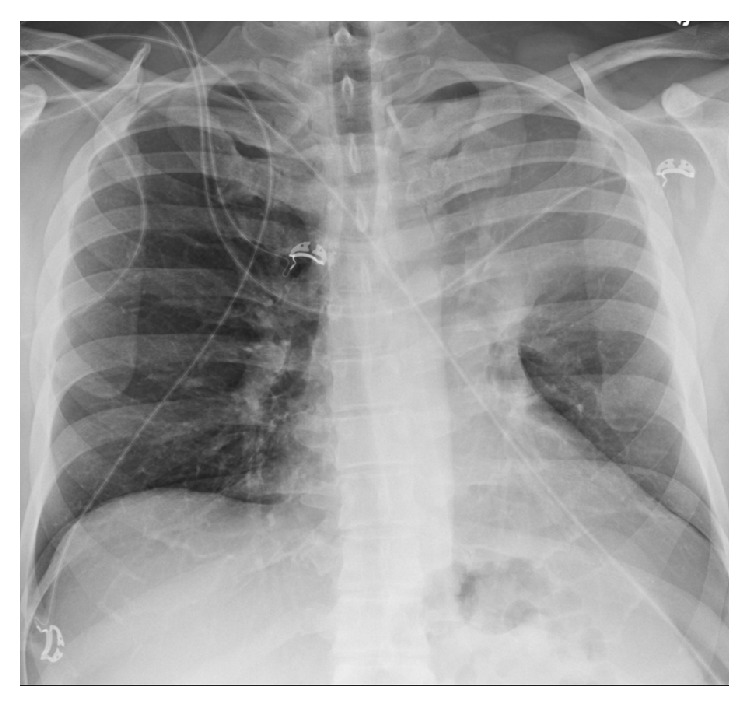
PA chest radiograph demonstrates complete left upper lobe atelectasis and the luftsichel sign.

**Figure 2:**
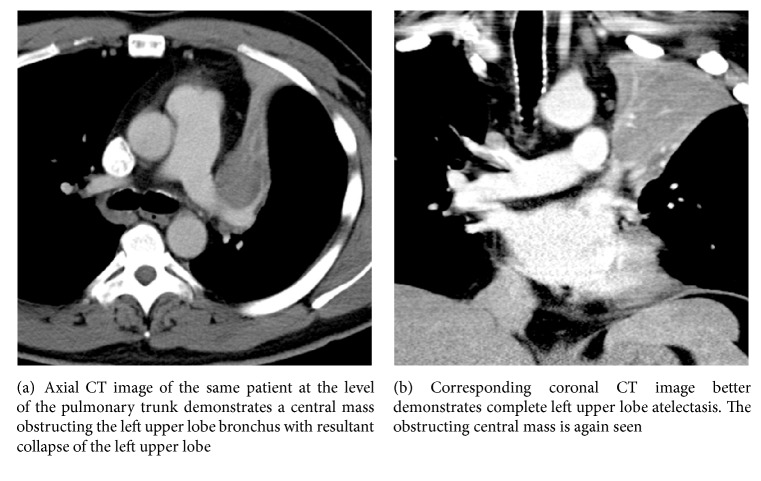


A 42-year-old male presented with nonproductive cough, shortness of breath, 15-pound weight loss, and night sweats for one month in duration. There was no history of smoking, upper respiratory symptoms, or chest pain. Physical exam showed shortness of breath and mildly decreased breath sounds in the left upper lung zone. Initial chest radiograph showed the luftsichel sign (i.e., complete atelectasis of the left upper lobe) and trace left pleural effusion (Figure 1). Subsequent CT scan showed complete left upper lobe atelectasis with a distinct central left upper lobe mass measuring 4.5 × 3.5 cm obstructing the left upper lobe bronchus (Figure 2). The patient eventually underwent further lab work, bronchoscopy, and PET-CT for further testing.

**Figure 3:**
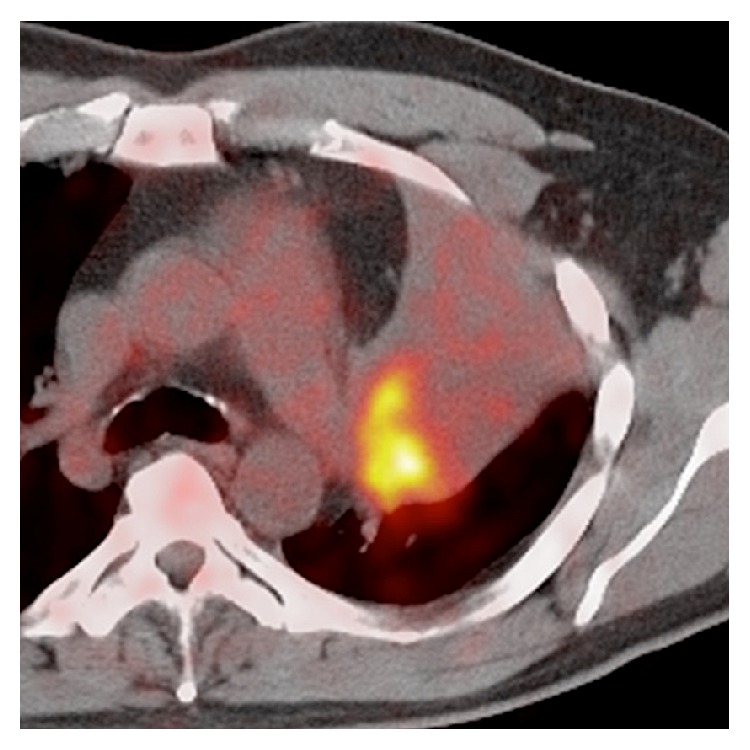
Axial PET/CT image demonstrates avid FDG uptake by the left upper lobe mass.

**Figure 4:**
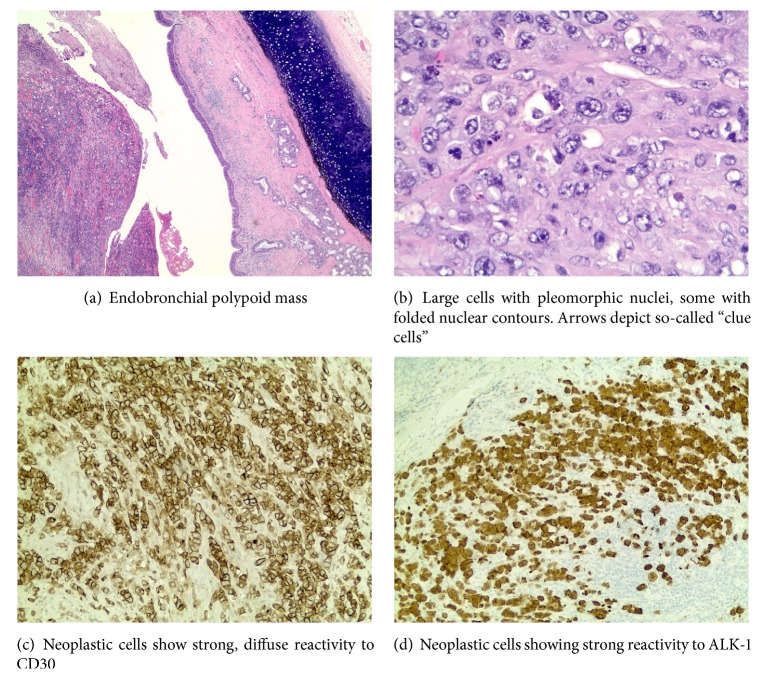


The PET-CT demonstrated focally increased metabolic activity within the left upper lobe which was favored to represent lung cancer or, less likely, metastatic disease (Figure 3). Bronchoscopy revealed a large tumor obstructing the left upper lobe segmental bronchus. Bronchoscopic biopsies of the mass showed neoplastic cells with large nuclei, scant cytoplasm, and vesicular nuclear chromatin, suggestive of a poorly differentiated malignant process. Due to lack of definitive immunohistochemical staining characteristics, additional percutaneous biopsy was performed which was indeterminate for malignancy. Eventually the patient underwent left pneumonectomy with final histology including immunohistochemistry demonstrating anaplastic large cell lymphoma positive for CD30, Ki-67, CD45, and ALK-1 (Figure 4). A full list of antibodies tested and results are listed in Table 1. An excised left hilar lymph node was free of tumor.

The patient recovered satisfactorily. A follow-up CT performed after 6 months showed no signs of recurrent disease.

## 3. Discussion

Imaging appearance of primary pulmonary non-Hodgkin lymphoma is varied. One retrospective study described the computed tomography findings in multiple cases of primary and secondary pulmonary lymphoma which included consolidation, ground-glass opacification, air-bronchograms, lymphadenopathy, CT-halo sign, lung nodules, reticular opacities, and pleural effusions [3]. In the majority of pulmonary non-Hodgkin lymphoma cases, patients presented with a combination of multiple CT findings. The most common combination of findings in primary and secondary non-Hodgkin's lymphoma included consolidation with air bronchogram, ground-glass opacities, and lymphadenopathy. As described, the CT findings are often nonspecific, and therefore could resemble various pathological processes. The differential diagnosis may include infectious processes, other neoplasms, inflammatory processes, or autoimmune processes. Interestingly, left upper lobe atelectasis, which is present in this case, seen on chest radiographs in the form of the luftsichel sign, is not a common imaging description in PPL. On the contrary, the luftsichel sign is almost always indicative of central primary lung cancer. Due to the variety and lack of specificity of the presenting imaging findings, definitive diagnosis requires tissue for histopathological examination.

In this case, the initial diagnosis of anaplastic large cell lymphoma was challenging. Ultimately, the resected specimens proved diagnostic. Morphologically, this neoplasm lacked typical glandular, squamous, or neuroendocrine differentiation characteristic of most primary lung cancers. Immunohistochemical studies further substantiated this morphologic impression by negative reactivity for pancytokeratin and CAM 5.2. The possibility of metastatic melanoma was effectively ruled out by negative reactivity for S-100 protein. The morphologic consideration of lymphoma was substantiated by patchy reactivity for CD45. The possibility of B-cell lymphoma was effectively ruled out by negative reactivity for CD20 and PAX5. Strong immunoreactivity for CD30 and ALK-1 are considered diagnostic in this morphologic and immunophenotypic context. Expressions of other T-cell markers are variable, as demonstrated in this case.

Anaplastic large cell lymphoma (ALCL) is a rare non-Hodgkin T-cell lymphoma characterized by large lymphoid cells with abundant cytoplasm, pleomorphic, horseshoe-shaped nuclei, and uniform CD30/Ki-1 expression [1, 4, 5]. ALCL was first described and reported by Stein et al. in 1985, as a neoplastic proliferation of lymphoid cells that are anaplastic in appearance, grow cohesively, invade lymph node sinuses, and consistently express CD30 [6, 7]. The morphology and patterns of tissue invasion commonly mimic those seen with non-small cell carcinoma or melanoma. Two types of ALCL with lung involvement have been described: primary pulmonary ALCL and secondary pulmonary ALCL. ALCL most commonly presents as a primary cutaneous lymphoma and may secondarily involve the lungs. Primary pulmonary ALCL is exceedingly rare and most often presents with mediastinal lymphadenopathy. Zhao et al. conducted a search of Medline and PubMed databases, in an attempt to identify all reported cases of primary ALCL of the lung, and found only 10 published cases between 1990 and 2015 [1].

ALCL is further subclassified based on positive or negative ALK protein expression. This protein is a tyrosine kinase encoded by a unique gene rearrangement. The most common gene rearrangement is t(2;5) which approximates the ALK gene located on chromosome 2 with the nucleophosmin NPM1 gene located on chromosome 5. Other ALK translocation partners occur with lesser frequency but most are involved with ALK protein upregulation. ALK is an important prognostic indicator for ALCL, as patients with ALK(+) staining tend to have a more favorable prognosis with a 5-year survival rate of 70–90%, compared to a 5-year survival rate of 40–60% in ALK(−) staining patients [8, 9]. Diagnosis of ALCL is based on the World Health Organization classification and includes typical histopathology and immunohistochemistry staining, with strong immunoreactivity for CD30.

There is no standardized treatment for ALCL of the lung; however, current first-line therapy includes anthracycline-based regimen, such as CHOP [10, 11].

## Figures and Tables

**Table 1:** Results of immunohistochemistry.

Antibody	Results
Pan cytokeratin	Negative in neoplastic cells
CAM 5.2	Negative in neoplastic cells
S-100	Negative in neoplastic cells
Vimentin	Positive, strong diffuse cytoplasmic staining
CD45	Negative to weakly positive in scattered neoplastic cells
CD20	Negative in neoplastic cells
PAX5	Negative in neoplastic cells
CD3	Negative in neoplastic cells
CD30	Positive, strong membrane and heterogeneous cytoplasmic staining
Alk-1	Positive, strong membrane and cytoplasmic staining
CD7	Negative in neoplastic cells
CD4	Negative for neoplastic cells
CD8	Weakly positive in neoplastic cells
CD10	Negative in neoplastic cells
CD15	Negative in neoplastic cells
CD56	Negative in neoplastic cells
CD68	Negative in neoplastic cells
MUM-1	Negative in neoplastic cells
Ki-67	Positive in greater than 90% of neoplastic cells
